# Construction of a poor prognosis prediction and visualization system for intracranial aneurysm endovascular intervention treatment based on an improved machine learning model

**DOI:** 10.3389/fneur.2024.1482119

**Published:** 2025-01-08

**Authors:** Chunyu Lei, Anhui Fu, Bin Li, Shengfu Zhou, Jun Liu, Yu Cao, Bo Zhou

**Affiliations:** ^1^Department of Neurosurgery, FuShun County Zigong City People's Hospital, Fushun, China; ^2^Department of Neurosurgery, Nanchong Central Hospital, Nanchong, China; ^3^Department of Neurology, The Third People's Hospital of Yibin, Yibin, China; ^4^Department of Neurosurgery, The Sixth People's Hospital of Yibin, Yibin, China

**Keywords:** improve machine learning models, intracranial aneurysm, intravascular intervention therapy, poor prognosis, visualization system

## Abstract

**Objective:**

To evaluate the clinical utility of improved machine learning models in predicting poor prognosis following endovascular intervention for intracranial aneurysms and to develop a corresponding visualization system.

**Methods:**

A total of 303 patients with intracranial aneurysms treated with endovascular intervention at four hospitals (FuShun County Zigong City People's Hospital, Nanchong Central Hospital, The Third People's Hospital of Yibin, The Sixth People's Hospital of Yibin) from January 2022 to September 2023 were selected. These patients were divided into a good prognosis group (*n* = 207) and a poor prognosis group (*n* = 96). An improved machine learning model was employed to analyze patient clinical data, aiding in the construction of a prediction model for poor prognosis in intracranial aneurysm endovascular intervention. This model simultaneously performed feature selection and weight determination. Logistic multivariate analysis was used to validate the selected features. Additionally, a visualization system was developed to automatically calculate the risk level of poor prognosis.

**Results:**

In the training set, the improved machine learning model achieved a maximum F1 score of 0.8633 and an area under the curve (AUC) of 0.9118. In the test set, the maximum F1 score was 0.7500, and the AUC was 0.8684. The model identified 10 key variables: age, hypertension, preoperative aneurysm rupture, Hunt-Hess grading, Fisher score, ASA grading, number of aneurysms, intraoperative use of etomidate, intubation upon leaving the operating room, and surgical time. These variables were consistent with the results of logistic multivariate analysis.

**Conclusions:**

The application of improved machine learning models for the analysis of patient clinical data can effectively predict the risk of poor prognosis following endovascular intervention for intracranial aneurysms at an early stage. This approach can assist in formulating intervention plans and ultimately improve patient outcomes.

## 1 Introduction

Intracranial aneurysms are protrusions resulting from congenital defects in the cerebral artery walls or increased intraluminal pressure, and they are a primary cause of subarachnoid hemorrhage. Clinically, these aneurysms often present with symptoms such as vomiting, severe headache, and visual field disturbances. Without timely intervention, ruptured aneurysms can lead to intracranial hemorrhage, posing significant threats to patient survival. The associated mortality and disability rates are notably high, leading to poor prognoses (1, 2).

Endovascular intervention is the primary treatment for intracranial aneurysms, offering advantages in reducing trauma for patients with ruptured aneurysms and promoting postoperative recovery. However, the factors influencing prognosis following this treatment remain unclear (3, 4). Traditional logistic regression models rely heavily on selected independent factors to predict patient outcomes, but they often miss critical clinical information, significantly reducing data utilization and overall predictive performance (5).

In recent years, machine learning, a branch of artificial intelligence, has shown great promise in the medical field. It can develop predictive models by analyzing clinical data and extracting case characteristics for accurate diagnosis and prognosis. Machine learning also allows for iterative improvements during validation, offering high efficiency and rapid results (6). Numerous studies have explored the use of machine learning models to predict outcomes following endovascular intervention for intracranial aneurysms. For instance, several studies have employed models such as support vector machines, random forests, and neural networks to enhance prediction accuracy and clinical decision-making (7–9).

Despite these advancements, there is still a lack of comprehensive research that integrates various machine learning techniques to improve the prediction of poor prognosis specifically for intracranial aneurysm endovascular intervention. Furthermore, existing studies often do not provide user-friendly visualization systems that can aid clinicians in interpreting the results and making informed decisions.

This study aims to bridge these gaps by evaluating the clinical value of using an improved machine learning model to predict poor prognosis in intracranial aneurysm endovascular intervention. Additionally, we aim to develop a visualization system to enhance the interpretability and usability of the predictive model for clinical practitioners. By doing so, we hope to provide a more robust and practical tool for improving patient outcomes.

## 2 Patients and methods

### 2.1 Patients

This study included 303 patients with intracranial aneurysms who underwent endovascular interventions at four hospitals (FuShun County Zigong City People's Hospital, Nanchong Central Hospital, The Third People's Hospital of Yibin, The Sixth People's Hospital of Yibin) from January 2022 to September 2023. The patients were divided into two groups based on their prognosis: the good prognosis group (*n* = 207) and the poor prognosis group (*n* = 96). Prognosis was evaluated at a 6-month follow-up using the Glasgow Outcome Scale (GOS) score (10), with scores of 4–5 indicating a good prognosis and scores of 1–3 indicating a poor prognosis. This retrospective study was approved by the Ethics Committee of Fushun County People's Hospital (Approval Number: 2023-077), and patient informed consent was waived. All patient data were anonymized and analyzed.

#### 2.1.1 Inclusion and exclusion criteria

Inclusion criteria: (1) head CT, MRI, cerebral angiography, and other examinations clearly for intracranial aneurysm; (2) endovascular interventional therapy; (3) complete clinical data.

Exclusion criteria: (1) pseudoaneurysm, non-aneurysmal subarachnoid hemorrhage; (2) other cerebrovascular diseases; (3) intolerance of surgical treatment.

### 2.2 Methods

#### 2.2.1 Information collection

Clinical data were collected for each patient, including age, gender, history of hypertension, preoperative aneurysm rupture status, Hunt-Hess classification, Fisher score, ASA classification, number of aneurysms, intraoperative use of etomidate, intubation status upon leaving the operating room, operation time, body weight, platelet count, platelet distribution, prothrombin time, partial activated prothrombin time, thrombin time, fibrinogen. Prior to model construction, all data were standardized using the *Z*-score to eliminate the influence of dimensional differences.

#### 2.2.2 Construction of improved model

The Automatic Feature Filtering and Weight Determination Integrated (AFFWDI) model is an innovative framework designed to enhance the prediction accuracy and generalization ability of machine learning models. The core of the AFFWDI model utilizes swarm intelligence algorithms, inspired by natural behaviors such as bird flocking and ant foraging, to solve optimization problems. The methodology of the AFFWDI model can be divided into two main steps.

##### 2.2.2.1 Feature screening process

(1) Initialization: Randomly generate a population of solutions, each representing a subset of possible features. (2) Adaptation Assessment: Assign a fitness value to each feature subset, usually based on its performance in a predictive model. In this study, the fitness is measured by the accuracy of cross-validation. (3) Search for Updates: Update each solution, i.e., a subset of features, according to the rules of the swarm intelligence algorithm. (4) Termination Conditions: Repeat the iterations until the termination conditions are met, such as reaching the maximum number of iterations or when the fitness value no longer shows significant improvement.

##### 2.2.2.2 Weight determination process

(1) Weight initialization: After determining the optimal subset of features, initial weights are assigned to these features. (2) Weight Optimization: The same swarm intelligence algorithm is used to optimize the weights, thereby improving the overall prediction performance of the model. (3) Synergistic Optimization: The optimization of feature subsets and weights is not conducted independently but synchronously, ensuring that the synergistic effect of feature screening and weight assignment is maximized.

#### 2.2.3 Improved intelligent algorithms

This study employs a swarm intelligence optimization algorithm to handle the complex tasks of feature screening and weight determination. To ensure superior global optimization capability, we introduce an improved Prairie Dog Optimization Algorithm (IPDO). The original Prairie Dog Optimization Algorithm (PDO) divides the behavior of prairie dogs into two stages: global exploration and local exploitation. However, previous studies have indicated a risk of the algorithm falling into local optima (11). To address this, the IPDO enhances exploration and convergence performance through Tent chaotic initialization and t-distribution perturbation variation. Detailed descriptions of these enhancements are as follows.

##### 2.2.3.1 Tent chaotic initialization

Tent mapping is a simple yet effective chaotic mapping method with excellent non-linear and traversal properties. By using Tent chaotic initialization, the algorithm can generate a more diversified and uniformly distributed solution space in the initial stage, which enhances the global search capability and prevents the algorithm from prematurely falling into local optima (12). During the algorithm's initialization phase, each prairie dog's location is no longer randomly generated but is determined by a Tent chaotic sequence, thus ensuring better diversity and coverage of the initial population.

##### 2.2.3.2 *t*-distribution perturbation variation

The t-distribution (Student's *t*-distribution) is a probability distribution whose shape is controlled by the degrees of freedom parameter. When the degrees of freedom are low, the *t*-distribution has a thicker tail, which allows for generating more significant variances with smaller probabilities when creating perturbations, thereby increasing the algorithm's ability to escape local optima (13). In the position updating stage, in addition to the traditional PDO position updating rules, a perturbation factor generated by the *t*-distribution is introduced to randomly perturb the position of an individual. This variation enhances the algorithm's local search ability, explores a broader search space to some extent, and improves the probability of finding the optimal global solution.

#### 2.2.4 Performance simulation testing of swarm intelligence algorithms

The optimization performance of the IPDO algorithm was tested using 23 standard test functions, each designed for minimization problems and varying in dimensions and complexity. Key characteristics of these functions include search space boundary range, function dimensionality, function category, and optimal solutions. The test functions were classified into unimodal (U) and multimodal (M) types. Unimodal functions assess local exploitation capability, while multimodal functions evaluate global exploration capability. To ensure a fair comparison, the population size was set at 30, the number of iterations at 200, and the average convergence curves were plotted after 30 repetitions.

### 2.3 Statistical analysis

SPSS 25.0 software was used to analyze the data, and the count data were expressed as [*n* (%)] and compared with *theχ*^2^-test; the normally distributed measure data were expressed as (x̄ ± s) and compared with *t*-test; the analysis of influencing factors was performed by multifactorial Logistic regression analysis, and the independent variables were entered into the regression equations by stepwise method. *P* < 0.05 was used to indicate statistically significant differences in two-sided tests.

## 3 Results

### 3.1 Performance test of improved group intelligence algorithm

**Figure 1:**
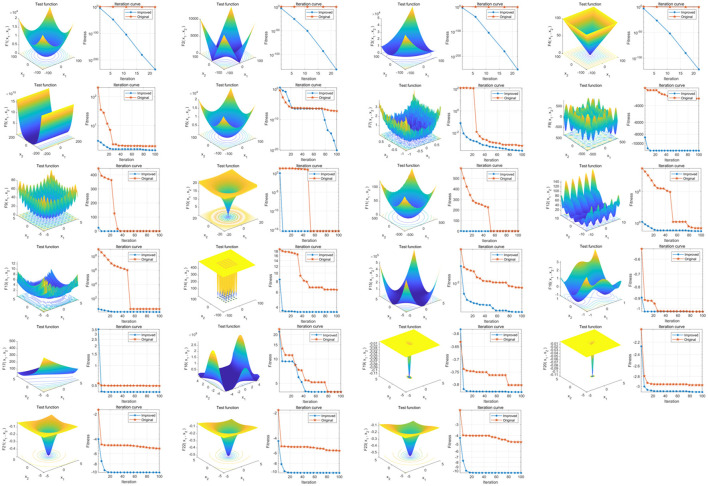
Comparison of optimization-seeking capability before and after PDO improvement. The three-dimensional surface plots in the figure show the two-dimensional search space of each benchmark function; the convergence curves show the convergence trend of the first solution in the first dimension of each benchmark function, and the trends of PDO and IPDO are compared. The red convergence curve in the figure corresponds to the original PDO algorithm, and the blue convergence curve corresponds to the improved IPDO algorithm.

The results show that the overall convergence speed and global optimization-seeking ability of the IPDO algorithm are significantly improved compared to the pre-improvement period (Figure 1).

### 3.2 Predictive modeling

#### 3.2.1 Model training

**Figure 2:**
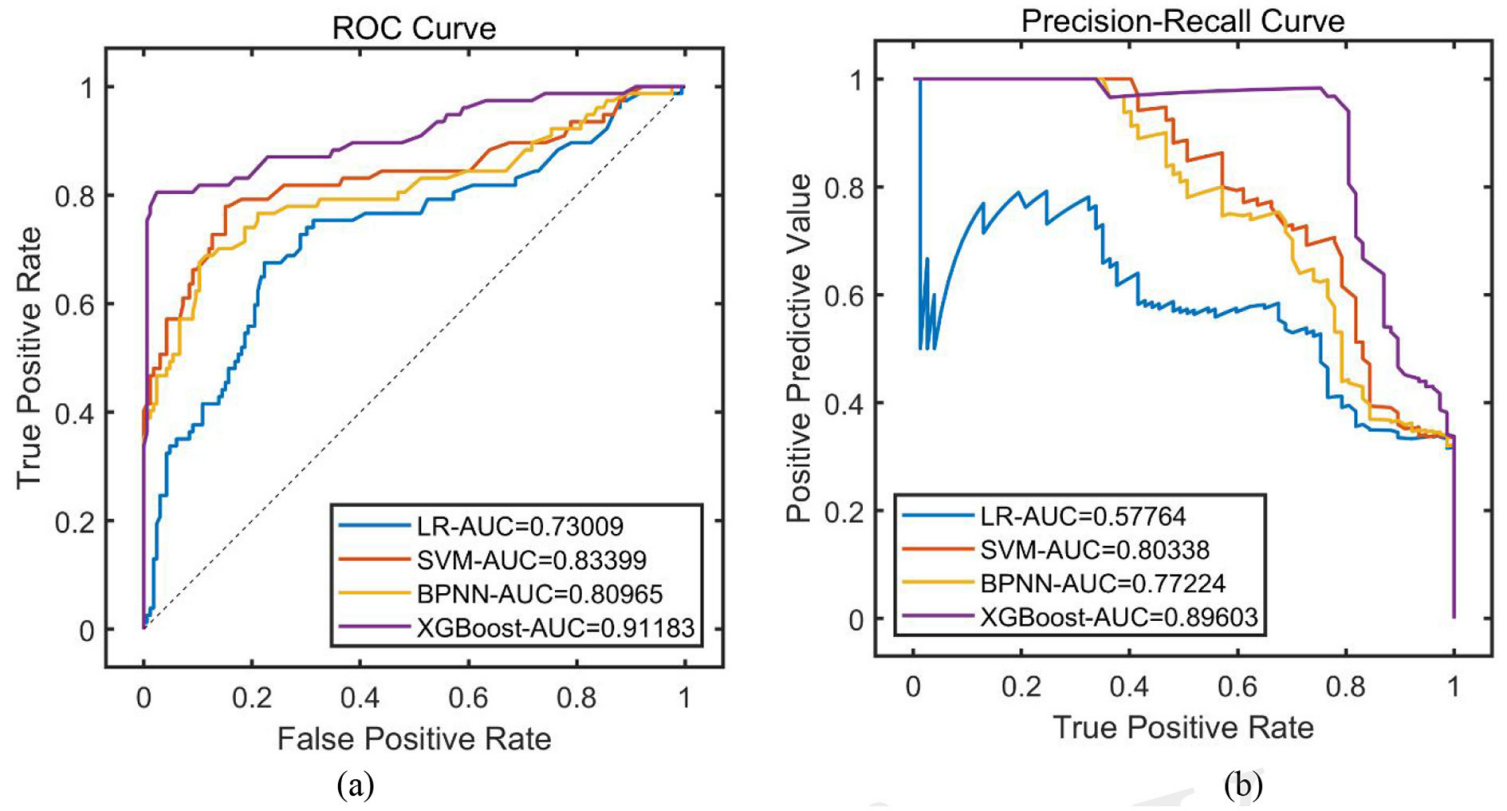
Training of each model (training set). **(A)** ROC curve; **(B)** PR curve.

Eighty percent of the dataset is randomly selected as the training set, cross-validation is executed, IPDO is utilized to find the optimal combination of features and hyper-parameters, and four types of base learners are selected; namely, LR, SVM, Back Propagation Neural Network (BPNN), and XGBoost, and the final model training results show that the AFFWDI model with XGBoost as the base learner performs optimally (Table 1, Figure 2).

**Table 1:** Training of each base learner based on simultaneous optimization.

**Base learning model (BLM)**	**PRE**	**SEN**	**SPE**	**ACC**	**F1**	**ROC-AUC**	**PR-AUC**
LR	0.7222	0.3377	0.9398	0.7490	0.4602	0.7301	0.5776
SVM	0.8542	0.5325	0.9578	0.8230	0.6560	0.8340	0.8034
BPNN	0.8409	0.4805	0.9578	0.8066	0.6116	0.8097	0.7722
XGBoost	0.9677	0.7792	0.9880	0.9218	0.8633	0.9118	0.8960

LR, Logistic Regression; SVM, Support Vector Machine; BPNN, Back Propagation Neural Network; XGBoost, eXtreme Gradient Boosting.

#### 3.2.2 Model testing

**Figure 3:**
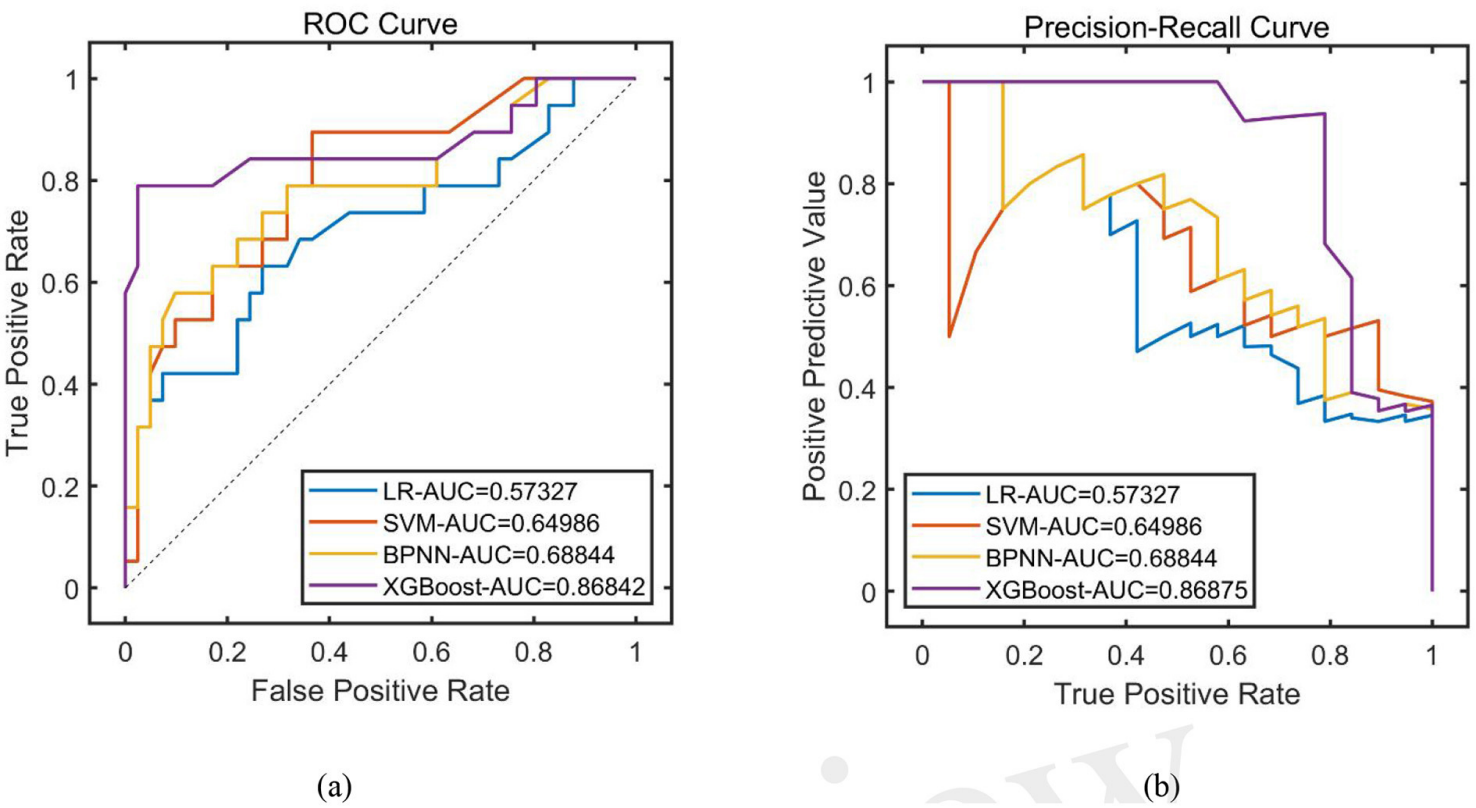
Comparison of prediction performance of models (test set). **(A)** ROC curve; **(B)** PR curve.

The remaining 20% of the dataset was used as a test set to examine the generalization ability of each model. The results showed that the AFFWDI model with XGBoost as the base learner had the best performance, and 10 variables were screened for age, comorbid hypertensive disorders, ruptured aneurysm preoperatively, Hunt-Hess classification, Fisher score, ASA classification, number of aneurysms, and intraoperative use of etomidate, intubation on leaving the operating room, and length of surgery (Table 2, Figure 3).

**Table 2:** Test set performance of each base learner based on synchronization optimization.

**Base learning model (BLM)**	**PRE**	**SEN**	**SPE**	**ACC**	**F1**	**ROC-AUC**	**PR-AUC**
LR	-	0.0000	1.0000	0.6833	-	0.6977	0.5733
SVM	0.8571	0.3158	0.9756	0.7667	0.4615	0.8017	0.6499
BPNN	0.8571	0.3158	0.9756	0.7667	0.4615	0.7811	0.6884
XGBoost	0.9231	0.6316	0.9756	0.8667	0.7500	0.8684	0.8688

LR predictions were all good prognoses and lost predictive value, so PRE and F1 values could not be calculated.

### 3.3 Feature revalidation

All 10 variables the improved machine learning model screened had statistically significant differences between the two groups (*P* < 0.05), coinciding with the logistic multifactor analysis results (Tables 3, 4).

**Table 3:** Univariate analysis.

**Variants**	**Good prognosis group *n* = 207**	**Poor prognosis group *n* = 96**	***t*/*c*^2^-value**	***p*-value**
Age	55.45 ± 10.34	58.28 ± 11.23	2.156	0.031
Hypertension			19.684	< 0.001
No	96 (46.38)	19 (19.79)		
Yes	111 (53.62)	77 (80.21)		
Preoperative aneurysm rupture			42.755	< 0.001
No	131 (63.29)	22 (22.92)		
Yes	76 (36.71)	74 (77.08)		
Hunt-Hess classification			34.438	< 0.001
I–II	145 (70.05)	33 (34.38)		
III–V	62 (29.95)	63 (65.62)		
Fisher score			25.309	< 0.001
1–2	194 (93.72)	70 (72.92)		
3–4	13 (6.28)	26 (27.08)		
ASA classification			8.187	0.004
I–II	131 (63.29)	44 (45.83)		
III–IV	76 (36.71)	52 (54.17)		
Number of aneurysms			5.643	0.018
1	166 (80.19)	65 (67.71)		
≥2	41 (19.81)	31 (32.29)		
Use of etomidate			5.494	0.019
No	171 (82.61)	89 (92.71)		
Yes	36 (17.39)	7 (7.29)		
Intubation			61.096	< 0.001
No	18 (8.70)	47 (48.96)		
Yes	189 (91.30)	49 (51.04)		
Surgical time/min	156.34 ± 21.99	40.23 ± 7.28	50.430	< 0.001

**Table 4:** Logistic multivariate analysis.

**Variants**	**β**	**SE**	**Wald χ^2^**	***P*-value**	***OR* value**	**95% CI**
Age	1.256	0.417	9.072	0.003	3.511	1.551–7.951
Hypertension	1.436	0.452	10.093	0.002	4.204	1.733–10.195
Preoperative aneurysm rupture	1.343	0.491	7.482	0.006	3.831	1.463–10.028
Hunt-Hess classification	1.411	0.485	8.464	0.004	4.100	1.585–10.608
Fisher score	1.293	0.406	10.142	0.002	3.644	1.644–8.075
ASA classification	1.277	0.451	8.017	0.005	3.586	1.481–8.679
Number of aneurysms	1.302	0.429	9.211	0.003	3.677	1.586–8.524
Use of etomidate	1.419	0.463	9.393	0.002	4.133	1.668–10.242
Intubation	1.391	0.406	11.738	0.001	4.019	1.813–8.906
Surgical time	1.274	0.403	9.994	0.002	3.575	1.623–7.876

### 3.4 Visualization system setup

**Figure 4:**
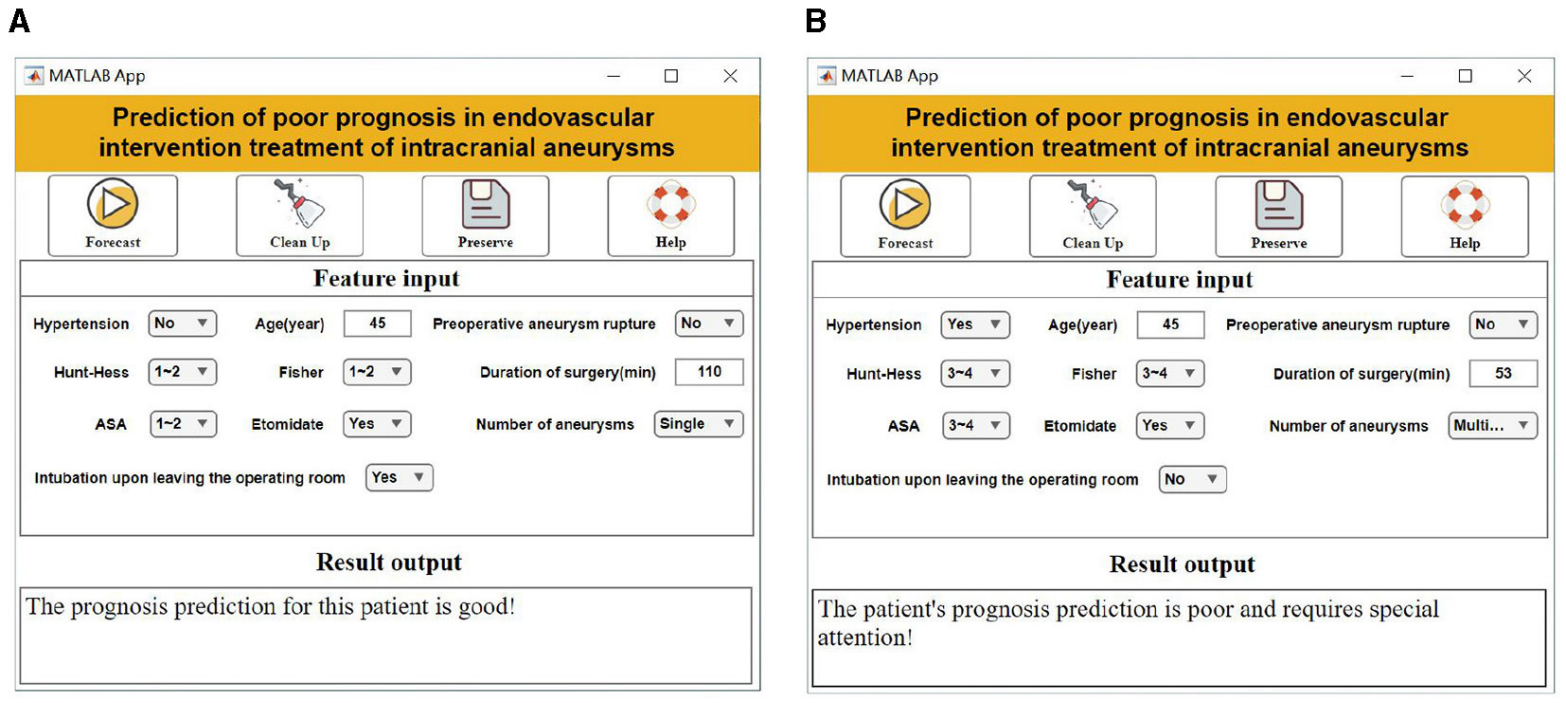
Visualization system interface display. **(A)** Good prognostic forecasting demonstration. **(B)** Demonstration of poor prognostic prediction.

In the application of the visualization system, the user only needs to enter 10 specific values in the “Characteristic Input” field, including “age, combined hypertension, preoperative aneurysm rupture, Hunt-Hess classification, Fisher score, ASA classification, number of aneurysms, intraoperative use of etomidate, intubation, length of surgery.” The system automatically calculates the risk of poor prognosis for a patient by assigning specific values to each of the 10 characteristics: age, Hunt-Hess classification, Fisher score, ASA classification, number of aneurysms, intraoperative use of etomidate, time of intubation, and length of surgery (Figure 4).

## 4 Discussion

Intracranial aneurysms are vascular abnormalities characterized by the abnormal bulging of intracranial artery walls, and their rupture is associated with high rates of disability and mortality (14, 15). Therefore, preventing aneurysm rebleeding is crucial. Endovascular intervention has increasingly been recommended as the preferred treatment for intracranial aneurysms due to its advantages such as reduced trauma, shorter operation times, and minimal damage to brain tissues. However, patients undergoing this procedure are susceptible to cerebral vasospasm and face a high rate of postoperative recurrence, both of which contribute to poor prognosis (16, 17). Consequently, analyzing the risk factors that influence the poor prognosis of endovascular intervention for intracranial aneurysms is vital for improving patient outcomes.

Machine learning has shown significant promise in enhancing clinical prediction efficacy by analyzing clinical data and applying specific algorithms to predict various outcomes (18, 19). By learning from multiple data modules, machine learning effectively identifies variables associated with patient outcomes, accurately predicts relevant risk factors, explores patterns, and builds mathematical models from complex data. It can also be iteratively calibrated during validation (20, 21). The Prairie Dog Optimization (PDO) algorithm, inspired by the behavior of prairie dogs, offers advantages such as easy implementation and balanced exploration and exploitation capabilities (22). However, it faces challenges such as slow convergence speed and low optimization accuracy. To address these issues, we implemented Tent chaotic initialization and *t*-distribution perturbation variation to initialize the population. Additionally, we incorporated suboptimal individual guidance strategies, natural enemy avoidance strategies, and adaptive probability threshold guidance strategies. These enhancements improve the algorithm's ability to avoid local optima and ensure higher solution accuracy and faster convergence speeds for the PDO algorithm (23).

In this study, we developed an improved machine learning model to predict poor prognosis in patients undergoing endovascular intervention for intracranial aneurysms. By adjusting relevant parameters for different algorithms and employing 5-fold cross-validation, we minimized the effect of randomness and prevented overfitting. Model performance on the test set was further enhanced through pruning, leading to the identification of 10 key variables: age, comorbid hypertension, preoperative aneurysm rupture, Hunt-Hess classification, Fisher score, ASA classification, number of aneurysms, intraoperative use of etomidate, intubation status upon leaving the operating room, and procedure duration.

The identified variables provide valuable insights into the factors influencing poor prognosis. For instance. Age: Older patients show decreased vascular elasticity and repair capabilities, increasing the risk of complications and recurrence post-intervention (24). Hypertension: Hypertension exacerbates damage to the intracranial vascular wall and alters hemodynamics, leading to higher risks during and after the procedure (25, 26). Preoperative Aneurysm Rupture: Ruptured aneurysms complicate the intervention due to fragile vasculature and increased aneurysm numbers, leading to higher recurrence and poor prognosis (27, 28). Hunt-Hess Classification and Fisher Score: Higher scores indicate severe intracranial hemorrhage and increased risk of complications like vasospasm and edema, which adversely affect outcomes (29–31). ASA Classification: Higher ASA scores reflect severe underlying conditions and lower surgical tolerance, impacting recovery and prognosis (32, 33).

Compared to traditional logistic regression models, our machine learning model leverages a broader range of clinical data and advanced algorithms to enhance predictive accuracy. Previous studies have utilized various machine learning techniques such as support vector machines and neural networks for similar purposes (7–9). However, our approach integrates the Prairie Dog Optimization (PDO) algorithm with enhancements like Tent chaotic initialization and t-distribution perturbation, which improve convergence speed and solution accuracy. The development of a visualization system based on our model allows clinicians to input specific patient data and receive immediate risk assessments and recommendations. This tool has significant potential for both public health research and clinical practice, aiding in early intervention and personalized treatment planning.

Although our study aims to construct a prediction and visualization system for poor prognosis in intracranial aneurysm endovascular treatment based on an improved machine learning model, we acknowledge several limitations.

Firstly, the predictive capability of our model is constrained by the quality and scale of the currently available dataset. A larger and more comprehensive dataset could potentially enhance the model's accuracy and stability. Secondly, our research focuses on specific treatment methods and populations. Future studies should consider a broader range of treatment methods and population factors to increase generalizability. Additionally, the interpretability and clinical applicability of the model require further optimization and improvement.

Future research can expand in several directions. Firstly, we can further optimize our machine learning model by exploring more advanced algorithms and technologies to enhance predictive performance and improve interpretability. Secondly, incorporating additional clinical variables and imaging features into the model could enhance the accuracy and comprehensiveness of prognosis prediction. Furthermore, integrating other advanced technologies, such as deep learning and natural language processing, could enrich the functionality and efficacy of the predictive model. Finally, integrating our system with actual clinical practice and conducting large-scale validation and application will ensure its effectiveness and reliability in real clinical settings.

By continuously refining our research methods and technologies, we are confident that future studies will provide more accurate and reliable support for prognosis prediction and clinical decision-making in intracranial aneurysm endovascular intervention treatment. This will offer greater hope and opportunities for patient treatment and recovery.

## 5 Conclusion

In summary, applying improved machine learning models to analyze patients' clinical data can help clinics predict the risk of poor prognosis following endovascular intervention for intracranial aneurysms at an early stage. This can assist in developing intervention programs to improve patient outcomes.

## Data Availability

The raw data supporting the conclusions of this article will be made available by the authors, without undue reservation.
